# Quadruplexes with a grain of salt: influence of cation type and concentration on DNA G4 stability

**DOI:** 10.1007/s00249-025-01772-w

**Published:** 2025-06-25

**Authors:** Anne Cucchiarini, Filip Kledus, Yu Luo, Václav Brázda, Jean-Louis Mergny

**Affiliations:** 1 Laboratoire d’Optique & Biosciences, Ecole Polytechnique, CNRS UMR7645 – INSERM U1182, Institut Polytechnique de Paris, 91120 Palaiseau Cedex, France; 2 Institute of Biophysics, Czech Academy of Sciences, Královopolská 135, 61200 Brno, Czech Republic; 3 National Centre for Biomolecular Research, Faculty of Science, Masaryk University, Kamenice 5, 62500 Brno, Czech Republic

**Keywords:** Cations, Ionic strength, G-quadruplex, Duplex-quadruplex competition, Halophile

## Abstract

G-quadruplexes (G4) are stabilized by intra-quartet hydrogen bonds stacking between quartets, as well as specific and non-specific ionic interactions. Cation effects on G-quadruplexes differ significantly from those on duplexes, and specific cation *coordination* is indeed required to stabilize G4 structures. Most studies so far involve “standard” concentrations of potassium or sodium cations because of their prevalence in human cells, but several other monovalent and divalent cations may promote quadruplex formation. In addition, ionic strength may be different in other organisms such as Halophiles: the intracellular cation (potassium) concentration in salt-loving organisms such as *Haloferax volcanii* can be extremely high. In this study, we first performed a bioinformatics analysis of G4 propensity in halophiles and analyzed the impact of altering ionic strength or ionic balance on G4 or hairpin duplex stability. We then present a detailed and quantitative assessment of salt effect on a variety of duplex and quadruplex sequences. Over a dozen different quadruplex and duplex sequences were investigated by FRET melting and UV melting experiments. In addition, changes in sodium/potassium balance possibly occurring in human cells have a modest effect on G4-duplex competition. We also confirm that lithium is rather a “G4-indifferent” than a G4-destabilizing cation.

**Supplementary Information:**

The online version contains supplementary material available at 10.1007/s00249-025-01772-w.

## Introduction

The cellular envirosnment is playing a crucial role in the formation of non-canonical nucleic acids. Apart from canonical DNA, nucleic acids strands can adopt a variety of structures such as G-quadruplexes (G4), cruciforms, triplexes or Z-DNA (Brazda et al. 2020; Bansal et al. 2022). Over the past decade, the research on non-B DNA structures has drastically increased due to their implications in key regulatory processes (*e.g.,* transcription or replication) and relevance for a significant fraction of the genome (Makova et al. 2024). To better understand their involvement at the cellular level, evidence regarding their formation and stabilization must be compiled. The formation of each of these structures is, of course, sequence-dependent, but is also stabilized by external factors such as ionic conditions.

G-quadruplexes or G4s are guanine-rich nucleic acids composed of four strands (DNA or RNA) and stabilized by non-covalent interactions: intra-quartet hydrogen bonds, π–π stacking between aromatic bases, and cation *coordination* (Spiegel et al. 2020). Indeed, specific cations may fit inside the quadruplex cavity and the nature of the cations itself has different effects on G4 stability and topology (Chen et al. 2021; Gajarsky et al. 2024). Potassium, which is octacoordinated, stabilizes G4s more than sodium, and the following general ranking has been proposed: Sr^2+^ > K^+^ > Ca^2+^ > NH_4_^+^, Na^+^, Rb^+^ > Mg^2+^ > Li^+^ ≥ Cs^+^ (Largy et al. 2016). Potassium—and to a lesser extent sodium—are the most popular cations for the biophysical and biochemical characterization of quadruplexes due to their prevalence in cells and their stabilizing effect. Typical K^+^ or Na^+^ concentrations are in the 100–150 mM concentration range, close to physiological conditions in humans. Yet, some organisms thrive under very different conditions, with a much higher salt concentration: for example, the halophile Archaea *Haloferax volcanii* requires a salinity of ~ 2.5 M of NaCl (Jevtić et al. 2019) and is maintaining a similar cationic concentration inside the cell, with ~ 2.5 M K^+^. There are indeed many species having optimal “physiological” ionic conditions different from human cells; yet, few studies have been dedicated to the prediction of G4s in halophiles, or to the effect of ionic strength on G4 *vs* duplex stability. In parallel, besides investigating changes in salt concentration, one may also consider changes in salt nature. This is physiologically relevant, as the sodium–potassium balance can be affected in human cells. K^+^ channels constitute the most diverse ion channel family group in the plasma membrane (Niemeyer et al. 2001; Comes et al. 2015), controlling physiological parameters such as cell volume, intracellular pH, and intracellular calcium levels (Spitzner et al. 2007). K^+^ channels play a crucial role as well in biological signaling by regulating key cellular processes such as proliferation, cell migration, and apoptosis (O’Grady and Lee 2005). These channels in healthy cell lines maintain the correct balance of Na^+^/K^+^, where intracellular concentration of potassium is higher than extracellular (~ 145 mM and ~ 5 mM, respectively) (Vodnala et al. 2019). In addition, these channels have been reported to have an oncogenic role (Niemeyer et al. 2001; Ousingsawat et al. 2007; Li and Xiong 2011; Comes et al. 2015) and are often overexpressed in some tumors. Indeed, it has been well documented that they are involved in aggressive cancers, including prostate, lung, colon, or breast cancer. Upon carcinogenesis, the cellular chemical environment may be altered, with a possible decrease in intracellular potassium concentration (Walker and Brown 1977; Tateishi-Karimata et al. 2018). Sugimoto and colleagues proposed that G4s may act as transcription inhibitors in healthy cells and that a decrease in potassium level within cancerous cells may enhance transcription.

For this reason, it may be interesting to investigate G4 formation under various salt conditions and to perform a bioinformatics search for G4s motifs in halophiles. Herein we describe and investigate the possible consequences of changes in ionic composition on G4 and canonical hairpin duplex stability. To this aim, we performed thermal studies of quadruplexes and duplexes under different ionic conditions. These studies may also be of interest for molecular biology, where G4 analysis using antibodies often involves permeabilization and changes in buffer, which may affect G4 stability.

## Materials and methods

### Halophile genomes

**Fig. 1:**
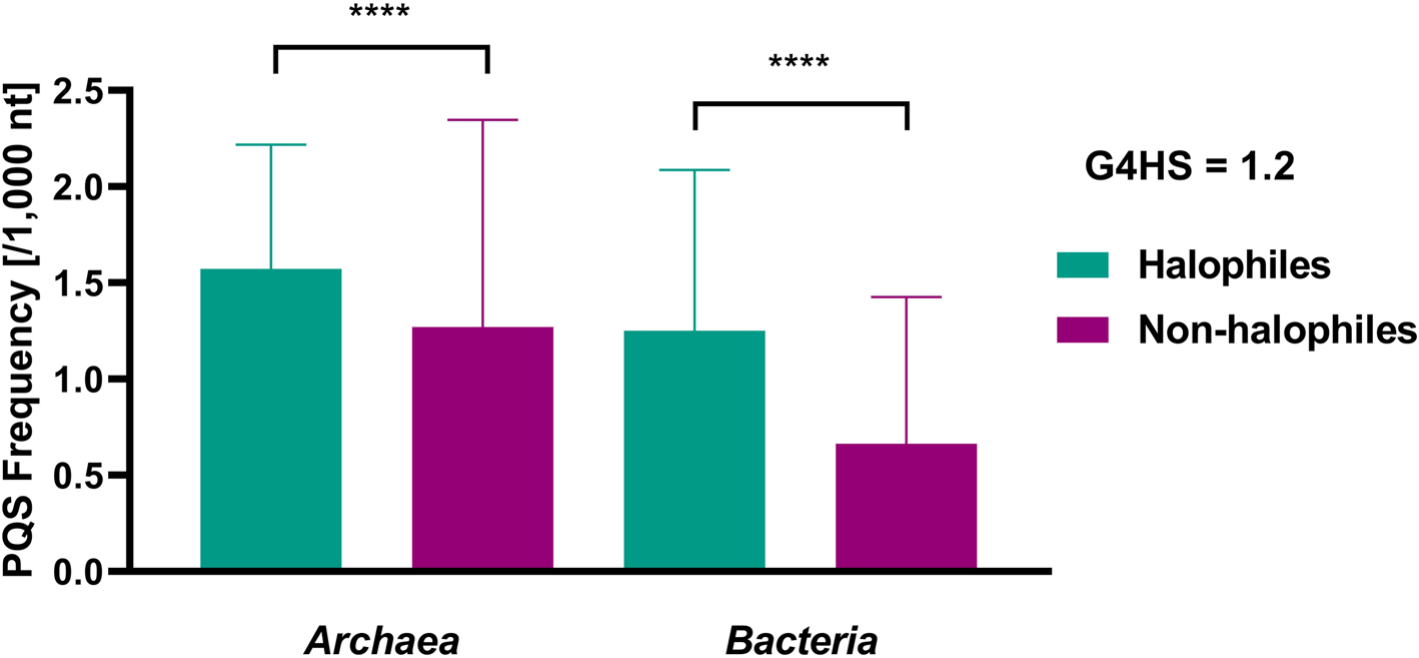
PQS frequency per 1000 bp in halophilic archaea (*n* = 162) and bacteria (*n* = 48, subset from taxa *GPB* and *Bacillota*) compared to representative non-halophilic archaeal (*n* = 123, kingdom *Methanobacteriati*) and bacterial genomes (*n* = 582, taxa *GPB* and *Bacillota*) (mean with SD). G4HS stands for G4Hunter score, the threshold used for this study. Statistical evaluations were made by Mann–Whitney test. Asterisks denote statistical significance: ****p < 0.0001

We analyzed 214 reference genomes of halophiles—162 archaeal genomes, mainly from phylum *Halobacteriota* (kingdom Methanobacteriati) and 52 bacterial genomes, mainly from phyla Pseudomonadota (class *Gammaproteobacteria (GPB)*) and *Bacillota* (Table 1, Fig. 1). For the analysis, we selected all available reference genomes (with assembly level complete) from the NCBI Genome database (downloaded on the 6th of January 2025) based on representative halophiles as reviewed by Oren in 2024 (Oren 2024). As a control group, we analyzed species from the same phylogenetic groups: for archaea, we analyzed 123 complete genomes of non-halophilic archaea (kingdom *Methanobacteriati*); for bacteria, we selected non-halophilic bacteria (582 complete genomes of *GBP* and *Bacillota*) from previously published dataset of G4Hunter analysis of bacterial domain (Bartas et al. 2019) (Supplementary Information S1).

**Table 1:** Statistical data of tested genomes

Domain	Seq	Median	GC %	PQS	Mean f	Min f	Max f
Halophilic archaea	162	3,320,769	64.31	848,013	1.57	0.18	3.45
Halophilic bacteria	52	3,862,281	52.97	280,421	1.38	0.04	5.01
Non-halophilic archaea	123	2,005,098	44.82	349,775	1.27	0.09	6.73
Non-halophilic bacteria	582	3,330,554	44.66	1,476,821	0.66	0.02	6.59

*Seq* total number of sequences, *Median* median length of genome sequences, *GC %* average GC content, *PQS* total number of predicted PQS, *Mean f* mean of predicted PQS per 1000 nt, *Min f* the lowest frequency of predicted PQS per 1000 nt, *Max f* the highest frequency of predicted PQS per 1000 nt

### G4Hunter searches

G4Hunter web server (Brázda et al. 2019) was used for the analyses of potential G-quadruplex sequences (PQS) in the full archaea and bacterial genomes with the default parameters (window of 25 nt and score threshold of 1.2). The results of analyses (number of PQS with different G4Hunter thresholds and PQS frequency per 1000 bp) for all analyzed genomes as well as statistical analyses for individual groups are provided in Supplementary Information S1.

### Statistical evaluation

The statistical significance of the observed difference in PQS frequencies between the datasets was determined using the Mann–Whitney U test. Statistical significance is shown by asterisks (*), where the number of asterisks correspond to the *p* value: *(*p* < 0.05), **(*p* < 0.01), ***(*p* < 0.001), and ****(*p *< 0.0001).

### Oligonucleotides

A set of model sequences, either forming G4s or hairpins (Supplementary Tables S2 and S3) were purchased from Eurogentec (Belgium) and delivered in lyophilized form. Stock solutions were prepared at 100 µM strand concentration in bi-distilled water and were kept at −20 °C. Concentrations were determined by absorbance at 260 nm using the Beer–Lambert law and the molar extinction coefficient provided by the manufacturer.

### Fluorescence spectroscopy (FRET melting)

The experiments were performed on pre-folded G-quadruplex and duplex structures: the oligonucleotides were heated at 95 °C for 5 min in the corresponding buffer (Supplementary Table S4) and slowly cooled down for 3 h. As previously reported (De Rache and Mergny 2015), the unfolding of a secondary structure can be monitored via FRET melting assay carried out in 96-well plates on a real-time PCR instrument (CFX96 Biorad system). This assay was performed with seven biologically relevant G4-forming sequences (six DNA and one RNA) and five synthetic duplex-forming sequences (Watson and Crick auto complementary with variable %GC content). All oligonucleotides mentioned above are double labeled with FAM (donor at the 5′ end) and TAMRA (acceptor at the 3′ end) to assess their stability in the presence of various ionic conditions. Each well was filled with 0.2 µM labeled DNA or RNA in a total volume of 25 µL. Solutions were prepared in multiple buffers and are detailed in Supplementary Table S4. The melting scan rate was 1 °C/min. The melting temperature is calculated by normalizing the fluorescence intensity and by determining the temperature for which the normalized signal is 0.5.

### Absorbance spectroscopy (UV melting)

The experiments were performed as described above, with non-modified oligonucleotides (three G4-forming DNA and one duplex DNA). The stability of these sequences was monitored via UV melting analysis carried out with a UV–visible absorbance spectrophotometer (Cary Eclipse Agilent). Each cuvette was filled with 3 µM DNA in a total volume of 500 µL of 10 mM LiCaco pH 7.2 supplemented with potassium (10 mM to 2 M).

### van t’Hoff analysis

The determination of model-dependent thermodynamics parameters first requires to convert the raw fluorescence melting curves into fraction folded as a function of temperature. This was made possible by calculating the upper and lower baselines of each melting curve (the T_*m*_ corresponding to the intercept between the median of the two baselines and the melting curve) thanks to Origin 2019. After getting the equations of the curves, one can calculate the fraction folded (or unfolded) θ. The association or dissociation constant (K_*a*_ or K_*d*_) can be calculated as follows:$\frac{\theta }{1-\theta }$. Then the van’t Hoff curve can be easily drawn, corresponding to ln*K* = f (1/T), with a restricted confidence interval between 5 and 95% of fraction folded.

## Results

### In silico analysis of G4 propensity in halophiles

Based on the recent review of halophilic prokaryotes (Oren 2024), we downloaded all fully assembled halophiles genomes (162 archaeal and 52 bacterial genomes). As a control group, we used fully assembled genomes of non-halophilic archaea (123 complete genomes of archaea from kingdom *Methanobacteriati)* and 582 non-halophilic bacterial genomes from the same groups as the halophilic bacterial genomes *(GBP* and *Bacillota).* The G4Hunter analyses with default settings detected 1,128,434 PQS in 214 halophilic sequences with an average PQS frequency of 1.53 per 1000 bp. The analysis of non-halophilic prokaryotes found 1,826,596 PQS in 705 genomes with an average PQS frequency of 0.77 per 1000 bp. The PQS frequency for halophilic organisms is, therefore, almost twice higher compared to the genomes in the same phylogenetic groups. To avoid the overrepresentation of these results based on abundance of bacterial genomes, we have counted results also for halophilic and non-halophilic archaea and bacteria independently (Table 1). The same trend of PQS abundance was observed for archaea, where PQS frequencies are 1.57 for halophiles compared to 1.25 for archaeal non-halophiles and for bacterial genomes in the same taxonomic groups (PQS frequencies halophiles/non- halophiles 1.27/0.66). Even if the average PQS frequency in halophiles was higher for Archaea, the highest PQS frequency among halophiles was found in a bacterium, *Salinibacter ruber* (5.01). One of the model archaea halophile organisms, *Haloferax volcanii,* has a PQS frequency of 1.55—close to the average PQS frequency of halophilic archaea.

The statistic evaluation of differences between halophilic and non-halophilic archaea and bacteria in the same taxonomical groups is shown in Fig. 1.

These results highlight the relevance of studying G4 formation under high salt conditions, as G4 motifs are even more prevalent in halophiles than in other prokaryotes.

### Sequence design and choice of experimental conditions

Our goal was next to test the effect of changes in cation balance or concentration on the stability of quadruplexes and hairpin duplexes. To measure the effect of salt changes, we first selected a set of model sequences, either forming G4s or hairpins (Supplementary Tables S2 and S3). Regarding buffer conditions, most experiments were performed with potassium and sodium given their prevalence in biological environments. Various total concentrations were chosen, either reflecting possible conditions in human cells or in halophiles. We also tested the effect of non-physiological cations such as Li^+^ as well as the effect of divalent ions such as MgCl_2_. The same set of sequences were chosen for all experiments below, which are organized depending on the salt change considered.

### Ionic strength

#### Adding lithium

**Fig. 2:**
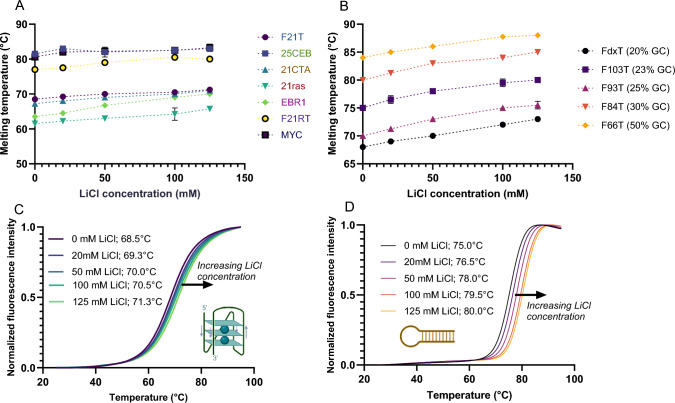
Melting temperature profiles of seven G4-forming sequences (**A**) and five hairpins duplexes (**B**), as a function of increasing concentrations of LiCl (KCl remains constant at 140 mM). Examples of melting profiles for a quadruplex (F21T) and a hairpin (F103T; 23%GC) are shown in panels (**C**) and (**D**), respectively. Pre-folded doubly labeled oligonucleotides (200 nM) are used to measure the fluorescence intensity as function of temperature

In this first series of experiments, we kept the potassium concentration constant and close to (human) physiological conditions and added variable concentrations of lithium from 0 to 125 mM. In other words, K^+^ concentration is constant but ionic strength varies. Lithium addition has a relatively modest effect on the stability of the tested G4s and duplexes, as shown in Fig. 2. The average ∆T_*m*_ between lowest and highest total ionic strength is + 3.5 °C and + 4.9 °C for G4s and hairpins, respectively. These results show a global *increase* in stability for all sequences, including G4s. This indicates that, contrary to popular belief, Li^+^ does not destabilize G4s; it even has a modest stabilizing effect. For duplexes, the trend was nearly independent on GC content (Supplementary Table S5).

#### Altering potassium concentration

**Fig. 3:**
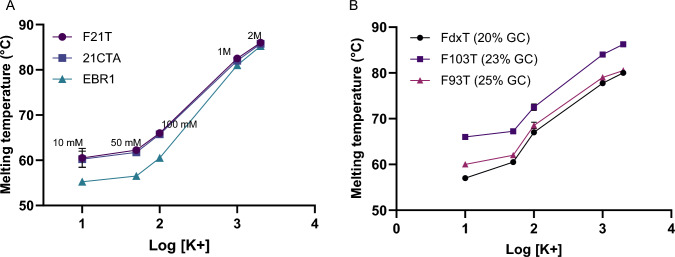
Melting temperatures of three selected G4 (**A**) and duplexes (**B**) as a function of the logarithm of KCl concentration (in mM). The concentration in potassium varied between 10 mM and 2 M. Only the sequences with low melting temperatures in 10 mM KCl were studied

The goal of this series of experiments was to assess the effect of high salt concentrations (in the molar range) on G4s, to reflect the situation possibly found in halophiles. While this change is expected to stabilize both duplexes and G4s, a comparative assessment is currently lacking. To explore this, we varied KCl concentration between 10 mM and 2 M, and determined T_*m*_ for the selected G4s and duplexes. Figure 3 illustrates the gap between molar and millimolar concentrations, both for G4s (Fig. 3A) and duplexes (Fig. 3B). The trend is quite similar: at low potassium concentrations and at a logarithmic scale, a modest slope can be observed, meaning that the change in stability is minimal (from 10 to 50 mM). As expected, the stability of duplexes and G4s drastically increases at 1 M, with average ∆T_*m*_ of + 13 °C and + 20 °C, respectively, between 100 mM and 1 M. Similar results were obtained by UV–Vis absorbance for the corresponding non-labeled G4 sequences 22AG, 26CEB, and22CTA as well as one hairpin (ds-lac) as shown in Supplementary Fig. S1. Of note, we checked the kinetic reversibility of these transitions, and recorded the heating and cooling profiles of 3 different quadruplexes under two cationic conditions. As can be seen in Supplementary Fig. S2, these curves are nearly superimposable at the chosen temperature gradient, meaning the profiles are indeed reversible or nearly-reversible. We also investigated whether this melting temperature was dependent on the scan rate (typically 1 C/min) by recording the melting profiles at different scan rates, between 0.3 and 3 C/min, at different ionic conditions. As can be seen in Supplementary Fig. S3, the apparent T_*m*_ determined at various scan rates were identical or very close. These observations allow us to conclude that the melting profiles indeed correspond to equilibrium curves.

**Fig. 4:**
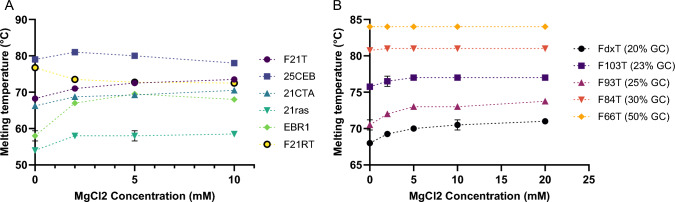
Melting temperature profiles of six G4-forming sequences (**A**) and five hairpins duplexes (**B**) as a function of increasing concentrations of MgCl_2_ (KCl concentration constant, 140 mM). Pre-folded double-labeled oligonucleotides (200 nM) were used to record fluorescence intensity as function of temperature

#### Adding dications

In this third series of experiments, we kept the potassium concentration constant, and added variable concentrations of MgCl_2_ from 0 to 10 mM for G4s and 0 to 20 mM for duplexes (Fig. 4**)**. Even if no absolute rule was found, general tendencies were identified. When starting from magnesium-free conditions, adding small amounts of MgCl_2_ (2 mM) tend to stabilize most structures, with a few exceptions: the two duplexes bearing the highest GC content and one RNA G4 (F21RT). Further increases in MgCl_2_ (up to 10 or 20 mM) had little or no effect on these structures.

### Ionic balance

In the next series of experiments, we kept the ionic strength constant and close to the physiological conditions relevant for human cells.

**Fig. 5:**
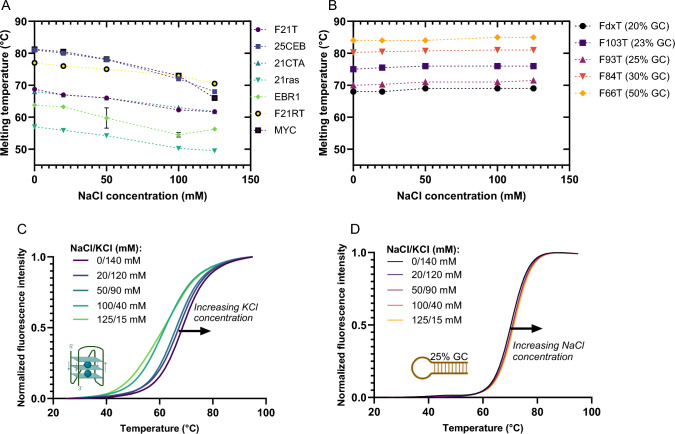
Melting temperature profiles of seven G4-forming sequences (**A**) and five hairpins duplexes (**B**). Each point represents a different ratio between NaCl and KCl, at a constant ionic strength. Bottom panels represent the melting curves obtained in those conditions for F21T and F93T (25%GC). Example of melting profiles for one G4 and one hairpin is shown in panels (**C**) and (**D**), respectively. Pre-folded double-labeled oligonucleotides (200 nM) are used to measure fluorescence intensity as a function of temperature

**Fig. 6:**
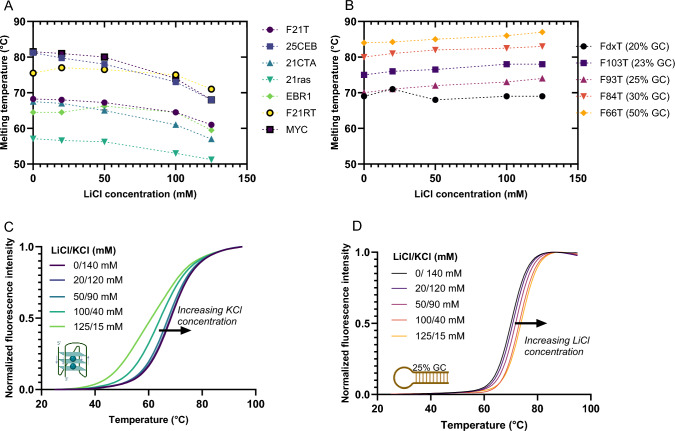
Melting temperature profiles of seven G4-forming sequences (**A**) and five hairpins duplexes (**B**). Each point represents a different ratio between LiCl and KCl, by keeping a constant ionic strength of 140 mM. Bottom panels represent the melting curves obtained in those conditions for F21T and F93T (25%GC). Pre-folded double-labeled oligonucleotides (200 nM) are used to measure the fluorescence intensity as a function of temperature

**Fig. 7:**
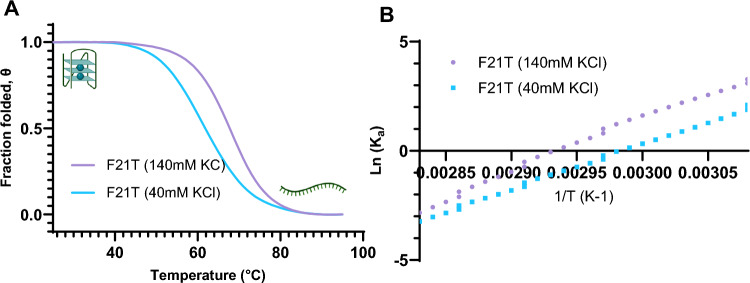
Example of calculation of fraction folded Theta θ (**A**) at 140 mM versus 40 mM KCl, needed to calculate the affinity constant K as a function of temperature (**B**), known as van’t Hoff’s curve

- 1) Changing the potassium/sodium balance
  We first analyzed the effect of sodium–potassium substitution on duplex and quadruplex stabilities. We previously reported the effect of this substitution on sequences capable of adopting both a hairpin and a G4s (Luo et al. 2024). G/C-rich sequences may indeed form a quadruplex or a competing hairpin structure based on G-C base pairing. The presence of cytosine in G4 loops did not prevent G4 folding or decrease G4 stability but increased the probability of forming a competing structure, either a hairpin or an intermolecular duplex, allowing us to design ‘Shape-shifters’, which respond to [Na^+^]/[K^+^] changes. In this study, while the [Na^+^]/[K^+^] balance is altered in a similar way, we analyze duplexes and quadruplexes independently, and results are shown in Fig. 5.
  Unsurprisingly, the nature of the cation has little or no effect on duplex stability, as shown in Fig. 5B and illustrated for one sequence for which the melting curves are shown in Fig. 5D**.** In contrast and according to our expectations, the T_*m*_ of all G4s depended on [Na^+^]/[K^+^] changes: thermal stability dropped by an average of 9.0 ± 3.6 °C depending on the sequence when replacing potassium by sodium (Fig. 5A). This effect is illustrated for the human telomeric motif F21T sequence in Fig. 5C.
- 2) Changing the potassium/lithium balance
  In the next series of experiments, rather than altering the [Na^+^]/[K^+^] balance, we decided to check the effect of the [Li^+^]/[K^+^] balance (Fig. 6). While this series of experiments may seem less physiological, there are, nevertheless, relevant for a number of molecular biology/sequencing experiments, in which K^+^ and Li^+^ conditions are compared. Our goal was to investigate to what extent potassium needed to be substituted by lithium to significantly destabilize a quadruplex. Results are summarized in Fig. 6A for all G4s considered, and melting profiles for the F21T sequence at different ratio are shown in Fig. 6C. As expected, stability drops (by 8.5 °C on average) when potassium is replaced by lithium. Nevertheless, it is clear that for this drop to be significant, a near-complete replacement has to be performed. Note that, as discussed before, this effect does not result from a “G4-destabilizing” property of lithium, but rather from the decreasing concentration of potassium. When lithium is added to a buffer in which potassium concentration is kept constant, no drop in stability is observed, as shown above.
  The results were quite different for duplexes, as illustrated in Fig. 6B. No clear destabilization was observed for any of the hairpins when replacing potassium by lithium. Interestingly, and surprisingly, a modest but significant stabilization was obtained for most of the hairpins.
  Overall, those results in Figs. 5, 6 confirm that K^+^ > Na^+^ in terms of G4 stability and that Na^+^ ≥ Li^+^. For duplexes, we could establish that Li^+^ ≥ Na^+^ ≥ K^+^, with much smaller effects.
- 3) Stability at physiological temperature
  The melting experiments we performed give us clues about the thermal stabilities of the structures considered. However, the melting temperature is not representative of the physiological conditions *in cellulo* and a ∆T_*m*_ cannot be directly converted into a difference in stability at physiological temperature, which is more relevant for cellular studies. All sequences were predominantly folded at 37 °C (meaning that their T_*m*_ was above 37 °C).
  To better understand and quantify the effect of sodium/potassium imbalance that may happen in cancerous cells, we decided to calculate the ∆G° of quadruplexes at a given temperature under different ionic conditions. We chose to compare two conditions, one expected to correspond to healthy cells ([KCl]_i_ = 140 mM), the other corresponding to cancerous or apoptotic cells in which potassium concentration has been reported to be severely reduced ([KCl]_i_ = 40 mM; [NaCl]_i_ = 100 mM). We selected three G4s (F21T, 21CTA, EBR1) for which the quality of the melting profiles allowed us an accurate determination of the fraction folded as a function of temperature under both conditions. These analyses are shown in Fig. 7 and Table 2. For all structures, the partial replacement of potassium by sodium ions also leads to a decrease in T_*m*_*,* as the melting temperature is systematically higher in pure potassium. Noticeably, higher potassium levels lead to a more enthalpy-driven folding (more exothermic) for all G4s. This high exothermic folding is compensated by a higher entropic component, meaning stronger intramolecular interactions and a higher rigidity.
  A change in T_*m*_ is not directly proportional to a change in equilibrium constant. To this aim, we calculated the ∆∆G° induced by cation change at two temperatures: the first, close to T_*m*_ and therefore very accurate and nearly assumption-independent, but far from physiological temperature and the second at physiological temperature which requires model-dependent extrapolations. Such calculations require that the melting profiles correspond to equilibrium curves; as discussed above, Supplementary Figs. S2–S3 indicate that the melting is kinetically reversible (T_*m*_ deduced from heating and cooling profiles are nearly identical) and that the T_*m*_ also minimally depends on the scan rate, between 0.3 and 3 °C/min. Both observations indicate the profiles analyzed reflect thermodynamic equilibrium. Of note, the ∆∆G° at high temperature are relatively small (0.1 to 0.7 kcal/mol or 0.4 to 3 kJ/mol), indicating that the changes in the equilibrium constant induced by changes in salt condition are modest.

**Table 2:** The thermodynamic parameters determined with a model-dependent (two-state) analysis, with the change in enthalpy and entropy ∆H and ∆S calculated from the van’t Hoff’s curves

Sequence	Salt conditions^*a*^	ΔH (kJ/mol)	ΔS (J mol^−1^ K^−1^)	ΔG_310.15 K_ (kJ/mol)	ΔG_343.15 K_ (kJ/mol)	K_ratio_	ΔTm _40–140_ (°C)	ΔΔG_40–140_ (kJ/mol)
F21T	KCl + NaCl	−172 ± 5	−514 ± 15	−12.6 ± 0.4	4.2 ± 0.4	0.4 ± 0.1	5.9 ± 1.0	5.4 ± 0.8 (37 °C)
	KCl	−203 ± 8	−597 ± 25	−18.0 ± 0.4	1.7 ± 0.4			3.0 ± 0.8 (70 °C)
EBR1	KCl + NaCl	−120 ± 3	−364 ± 11	−7.6 ± 0.4	4.6 ± 0.8	0.7 ± 0.1	7.9 ± 1.4	4.6 ± 0.4 (37 °C)
	KCl	−158 ± 3	−471 ± 8	−12.0 ± 0.4	3.8 ± 0.4			1.3 ± 0.4 (70 °C)
21CTA	KCl + NaCl	−171 ± 2	−508 ± 5	−13.8 ± 0.4	2.9 ± 0.4	0.8 ± 0.1	2.2 ± 1.3	3.3 ± 0.8 (37 °C)
	KCl	−201 ± 9	−592 ± 26	−17.2 ± 1.3	2.5 ± 0.4			0.4 ± 0.1 (70 °C)

Changes in free (Gibbs) energy were calculated at 37 °C (310.15 K) and 70 °C (343.15 K) with the following equation: $\Delta G=\Delta H-T\Delta S$, and assuming that ∆C_p_ = 0. The values correspond to the average of two replicates. ^*a*^: “KCl + NaCl” corresponds to 40 mM KCl + 100 mM NaCl. “KCl” corresponds to potassium only (140 mM)

## Discussion

G-quadruplexes are important non-canonical DNA structures found in various genomes from viruses to eukaryotes and including prokaryotes, both bacteria and archaea, playing crucial roles in various biological processes. Their presence is linked, for example, to gene regulation, thermal adaptation, and survival in extreme environments (Waller et al. 2016; Bartas et al. 2019). G4s are also implicated in pathogenicity, antibiotic resistance, and antigenic variability, suggesting their potential as biotargets for antibiotic development (Shitikov et al. 2022). We previously found that G4-forming sequences are present also across all archaeal species, with significant frequency variations and an average PQS frequency of 1.21 PQS/kbp (Brázda et al. 2020). Interestingly, when considering only halophilic archaea, we found a significantly higher average PQS frequency (1.57). A similar observation was found in bacteria: even if some bacterial groups have even higher PQS frequencies (Bartas et al. 2019), the comparison of the bacteria from the same taxonomical groups shows a statistically significant higher abundance of PQS in halophiles genomes. This higher abundancy of G4-prone motifs in halophilic bacteria and archaea could be the by-product of a higher average GC% in these species (Table 1) as G4 density is weakly correlated to GC% (Bartas et al. 2019; Bohálová et al. 2021).

Next, we investigated the effect of cations on G4s and duplex stability. The first experimental part studies the changes in total ionic strength, which is of interest for salt-loving organisms. After showing that halophiles tend to harbor more candidate G4 sequences than other prokaryotes, we demonstrated that an increase in salt concentration is associated with an increase in stability, as expected. A series of experiments analyzed the effect of adding lithium. This cation is sometimes referred to as a “G4 destabilizing agent”. We show here that adding lithium does not lead to a decrease in T_m_, as long as other G4-stabilizing agents are present. “G4 destabilizing conditions” rather correspond to conditions in which the buffer is devoid of stabilization cations (*e.g.*, sodium or potassium), but this does not mean that the cation replacing it (Lithium) is actively destabilizing. Lithium should rather be considered as a “G4-indifferent” cation, that will fail to stabilize a quadruplex if no favorable cations are present, but has no detrimental effect if such cations (potassium and, to a lesser extent, sodium) are present. We actually observed a limited *increase* in stability for G4s upon LiCl addition.

Higher than usual ionic concentrations may be relevant for a number of species. Salt-loving prokaryotes, archaea and bacteria have been reported. As emerged from our initial bioinformatical analyses of G4 content, halophile genomes exhibit a high density of PQS, exceeding the average density observed across in prokaryotes. Finding the right ionic conditions in vitro to mimic the conditions found in some halophiles may actually be difficult, as very high salt concentrations have been reported in some species of *Archaea*. These unicellular prokaryotes are often extremophile microorganisms. While they share common features with eukaryotic cells, like similarities in enzymes involved in replication, transcription and translation (Andrei et al. 2012), they may live under very different conditions. Halophilic archaea can tolerate salt concentration of up to 5 M and have developed mechanisms to counter the effect of such high salinity. *Haloferax volcanii,* a well-study halophilic archaea, is cultivated in 2.5 M NaCl (Jevtić et al. 2019) and maintains an intracellular ionic strength in the same range. The stability of the G4 motifs studied here was very high under these conditions, with T_*m*_ above 80°C. Interestingly, as can be seen in Supplementary Fig. S4, changes in salt concentration had little or no effect on quadruplex topology for two quadruplexes (22AG and 22CTA) but lead to significant changes in CD spectra for F21CTAT—an increase in NaCl concentration led to a more “anti-parallel like” spectra. This observation suggests that changes in ionic strength not only affect stability but also have an effect on folding (and most favorable topology) of some, but not all, of the G4-forming sequences.

The second part of the manuscript was dedicated to the study of cation balance, in which the ionic strength is kept constant, but the nature of the cation may vary. This series of experiments is no longer relevant for halophiles but may be of interest when comparing intra- and extra-cellular conditions, or when trying to mimic pathological situations in which K^+^ channels are deregulated. Unsurprisingly, G4s were found to be more sensitive to the nature of the cation than duplexes, which require only “nonspecific” cationic binding, as found for triplexes (Kim et al. 2015). Specific binding sites constitute a unique property of G-quadruplexes: cations such as K^+^ are dehydrated and coordinated within the G4 cavity and specific binding is occurring. This binding mode contrasts with the general and non-specific condensation of ions around DNA, which is less dependent on the nature of the cation. This condensation is explained by the polyelectrolyte effect, as each phosphate group bears a negative charge, and valid for most nucleic acid structures considered and independent of GC content for duplexes (Olejko et al. 2022). A change in cation balance may occur within cells due to the abnormal expression of potassium channels in some cancers. This allegedly leads to a decrease in intracellular potassium, down to values as low as 60 mM, and compensated by a concomitant increase in sodium levels. Unsurprisingly, this change alters G4 stability, but to a limited extent: Tm changed by 6.9 C in average for parallel G4s and the equilibrium constant by a factor of only ~ 0.7, while the stability of a duplex is minimally affected. Therefore, changes in cation balance possibly occurring in human cells have a modest effect on G4-duplex competition.

A change in cation balance may also be artificially induced by an experimenter, when considering “favorable” *vs* “unfavorable” conditions for G4 formation for molecular biology, sequencing or imaging experiments. This is often achieved by replacing potassium with lithium. We show here that G4 formation is still possible under Li^+^ conditions, as long as traces of potassium are present: even when T_*m*_ is decreased, the quadruplex can still form. Therefore, to really obtain unfavorable conditions, one has to severely reduce K^+^ concentration.

Both series of experiments relied on the same model sequences. The G4 sequences chosen may be considered as biologically relevant as they are found in oncogenes, telomeres, and parasites. FRET melting was chosen to evaluate and quantify stabilities: multiple conditions could be tested in a high-throughput fashion and over a short period of time. FRET melting is also of interest because of the low volume required (25 µL in most cases) and the low concentration needed (~ 200 nM) which disfavored competing equilibria with intermolecular reactions, therefore facilitating analysis. Cost considerations of dual-labeled oligonucleotides prevented us from testing hundreds of sequences though: a dozen of sequences were analyzed here. The results obtained with these modified oligonucleotides were confirmed by UV-absorbance melting experiments for a few unmodified sequences.

Overall, we provide quantitative figures on how quadruplex stability is affected by changes in ionic strength or cation balance. These results may be of interest for those working on halophiles, where cation concentration is much higher. The next step will be to analyze G4 formation and content in different types of halophiles, such as strict halophiles, which require high salt concentrations for growth (1.5–5 M) and are primarily found in hypersaline environments like salt lakes, salt pans, and saline soils.

## Supplementary Information

Below is the link to the electronic supplementary material.

Supplementary file1 (XLSX 100 KB)

Supplementary file2 (DOCX 1013 KB)

## Data Availability

All data used for this article are provided in the sup information, excel file, figures and tables.
