# Supplementary material for: Quadruplexes with a grain of salt: influence of cation type and concentration on DNA G4 stability
Source: Eur Biophys J. 2025 Jun 25;54(8):589–99. doi: 10.1007/s00249-025-01772-w (PMC12678505; doi:10.1007/s00249-025-01772-w)
Supplement: Supplementary file 2 — Supplementary file2 (DOCX 1013 KB) [file 249_2025_1772_MOESM2_ESM.docx]

**Quadruplexes with a grain of salt: influence of cations on DNA G4 stability**

Anne Cucchiarini, Filip Kledus, Yu Luo, Václav Brázda, Jean-Louis Mergny

**Supplementary information**

**Supplementary Tables:**

- S1: List of archaeal and bacterial genomes and the results of the G4Hunter analyses for individual genomes and statistics for archaeal and bacterial halophiles and non-halophiles (provided as an independent file in Excel format)
- S2: Quadruplexes used in thermal studies
- S3: Hairpins duplexes sequences
- S4: Table of ionic conditions
- S5: Slopes for duplexes in each condition

**Supplementary Figures :**

- S1: UV melting curves of 3 G4s and 1 duplex
- S2: Heating and cooling UV melting curves of 3 G4s
- S3: Effect of the melting scan rate (between 0.3 and 3°/min)
- S4: Effect of the increase in ionic strength on topology (sodium)
- S5: Effect of the increase in ionic strength on topology (potassium)

**Suplementary Table S2:** List of G4 sequences used for FRET melting (top) and UV-absorbance melting (bottom) studies.

| **Name** | **Sequence 5’ FAM – 3’ TAMRA** | **Topology** |
| --- | --- | --- |
| F21T | GGGTTAGGGTTAGGGTTAGGG | Hybrid |
| 25CEB | AGGGTGGGTGTAAGTGTGGGTGGGT | Parallel |
| 21CTA | GGGCTAGGGCTAGGGCTAGGG | Antiparallel |
| MYC | TTGAGGGTGGGTAGGGTGGGTAA | Parallel |
| 21ras | AGGGCGGTGTGGGAAGAGGGA | Parallel |
| EBR1 | GGGCAGGGGGTGATGGGGAGGAGCCAGGG | Parallel |
| F21RT | AGGGUUAGGGUUAGGGUUAGGG | RNA parallel |
| **Name** | **Sequence 5’-3’ (non-labeled)** | **Topology** |
| 22AG | AGGGTTAGGGTTAGGGTTAGGG | Hybrid |
| 22CTA | AGGGCTAGGGCTAGGGCTAGGG | Antiparallel |

**Suplementary Table S3:** List of duplexes (hairpin) sequences used for FRET melting (top) and UV-absorbance melting (bottom) studies.

| **Name** | **Sequence 5’ FAM – 3’ TAMRA**  **(X= PEG)** | **%GC** |
| --- | --- | --- |
| FdxT | TATAGCTATXTATAGCTATA | 20 |
| Fdx84T | AATCGATTGCATXATGCAATCGATT | 30 |
| Fdx66T | ATCAGTGCTCGAXTCGAGCACTGAT | 50 |
| Fdx103T | AATGATGAATCATXATGATTCATCATT | 23 |
| Fdx93T | ATCTTTAAACGTXACGTTTAAAGAT | 25 |
| **Name** | **Sequence 5’-3’ (non-labeled)** | **%GC** |
| Ds-lac | GAATTGTGAGCGCTCACAATTC | 45 |

**Suplementary Table S4:** Different ionic conditions and buffers used.

| **Type of experiment** | | **Buffer** | ***Cation* added** |
| --- | --- | --- | --- |
| **Ionic strength** | Lithium addition | 10 mM lithium cacodylate, pH 7.2,  140 mM KCl. | LiCl, 0 to 125 mM |
|  | Altering Potassium concentration | 10 mM lithium cacodylate, pH 7.2 | KCl, 10 mM to 2M |
|  | Adding dications | 10 mM lithium cacodylate, pH 7.2 | MgCl2, 0 to 10-20mM |
| **Ionic**  **balance** | Potassium/Sodium | 10 mM lithium cacodylate, pH 7.2 | KCl from 140 to 15 mM – NaCl from 0 to 125 mM |
|  | Potassium/Lithium | 10 mM lithium cacodylate, pH 7.2 | KCl from 140 to 15 mM – Cl from 0 to 125 mM |
| **Stability at physiological temperature** | | 10 mM lithium cacodylate, pH 7.2 | - 100 mM NaCl, 40 mM KCl - 140 mM KCl |

**Suplementary Table S5:** Calculation of the slopes (changes in T*_m_* as a function of mM salt added) using GraphPad Prism 10 and a simple linear regression.

| Slope for each condition/duplex (°C/mM) | FdxT | F103T | F93T | F84T | F66T |
| --- | --- | --- | --- | --- | --- |
| **LiCl addition** | **0.04** | **0.04** | **0.04** | **0.04** | **0.03** |
| **KCl *vs* NaCl** | **0.01** | **0.01** | **0.01** | **0.01** | **0.01** |
| **LiCl *vs* KCl** | **-0.006** | **0.02** | **0.03** | **0.02** | **0.02** |
| **MgCl_2_ addition** | **0.13** | **0.05** | **0.13** | **0.007** | **0.0** |

**Figure S1**

**
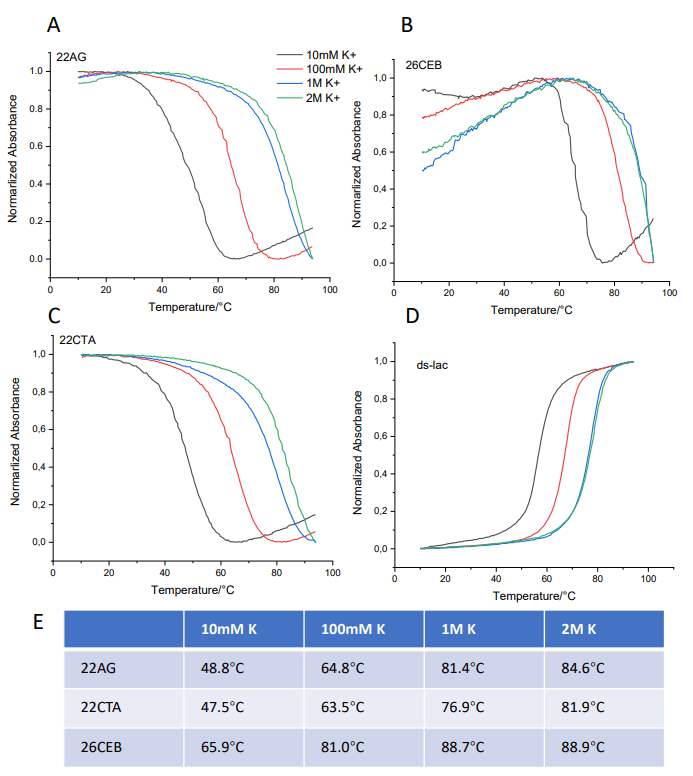
**

**Figure S1**. UV melting curves of 3 quadruplexes (22AG, 26CEB, 22CTA) (**A-C**) and 1 duplex (ds-lac) (**D**) sequences obtained at 295 and 260nm respectively, between 10 and 95°C. Melting temperatures determined from the curves at different concentrations of potassium (**E**).

**Figure S2**


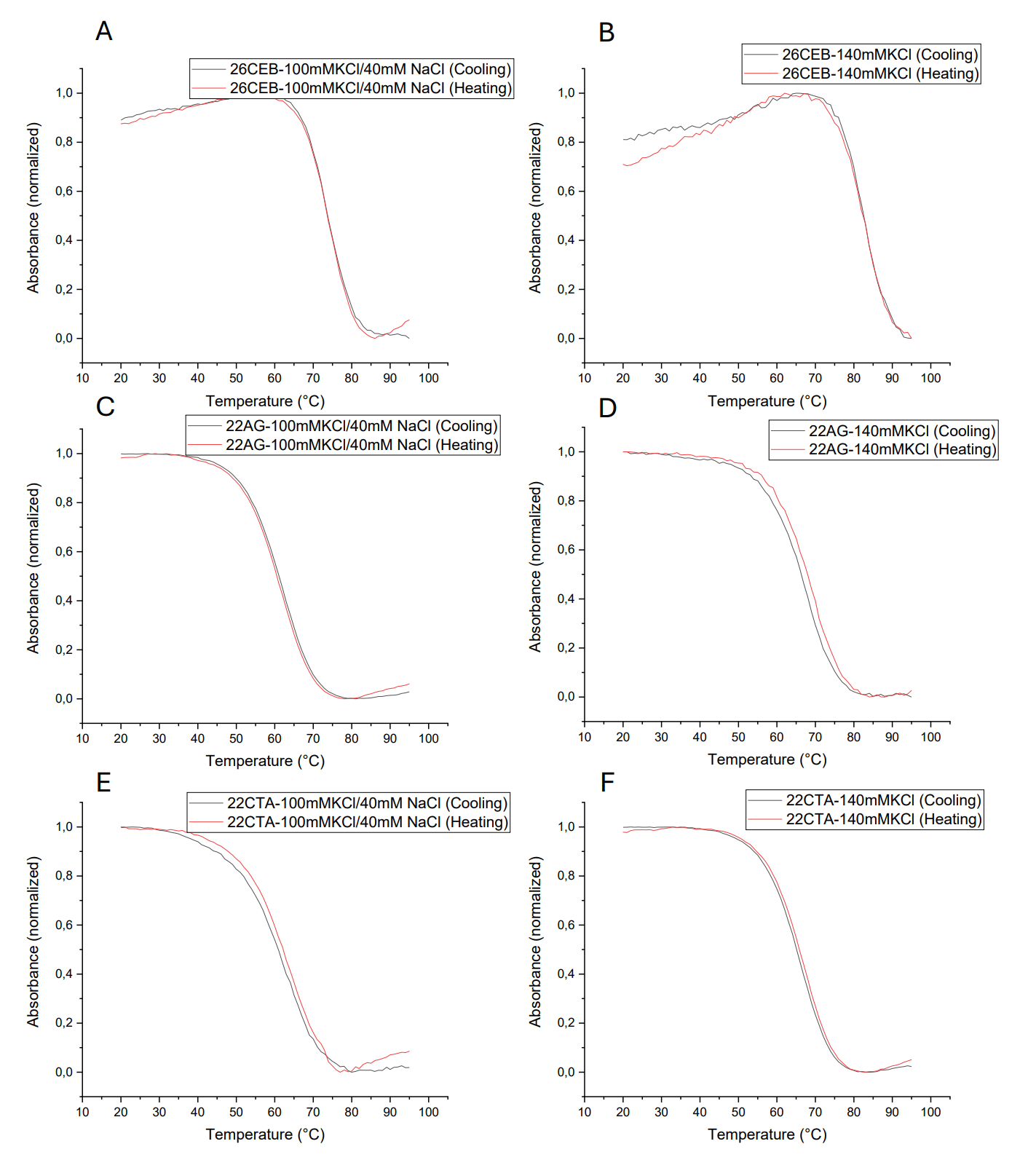


**Figure S2**. Heating and cooling UV-melting curves of 3 quadruplexes (26CEB, 22AG, 22CTA) in 10 mM licaco (pH 7.2), supplemented with 100 mM KCl + 40 mM NaCl **(A-C-E)** or 140 mM KCl **(B-D-F).** The oligonucleotides were annealed in the corresponding buffer and directly in the spectrometer (20 to 95°C, 20°/min, 5min), then the cooling curves (in black) were recorded (95 to 20°C, 0.5°/min). Finally, the heating curves (in red) were recorded (20 to 95°C, 0.5°/min).

**Figure S3**

**
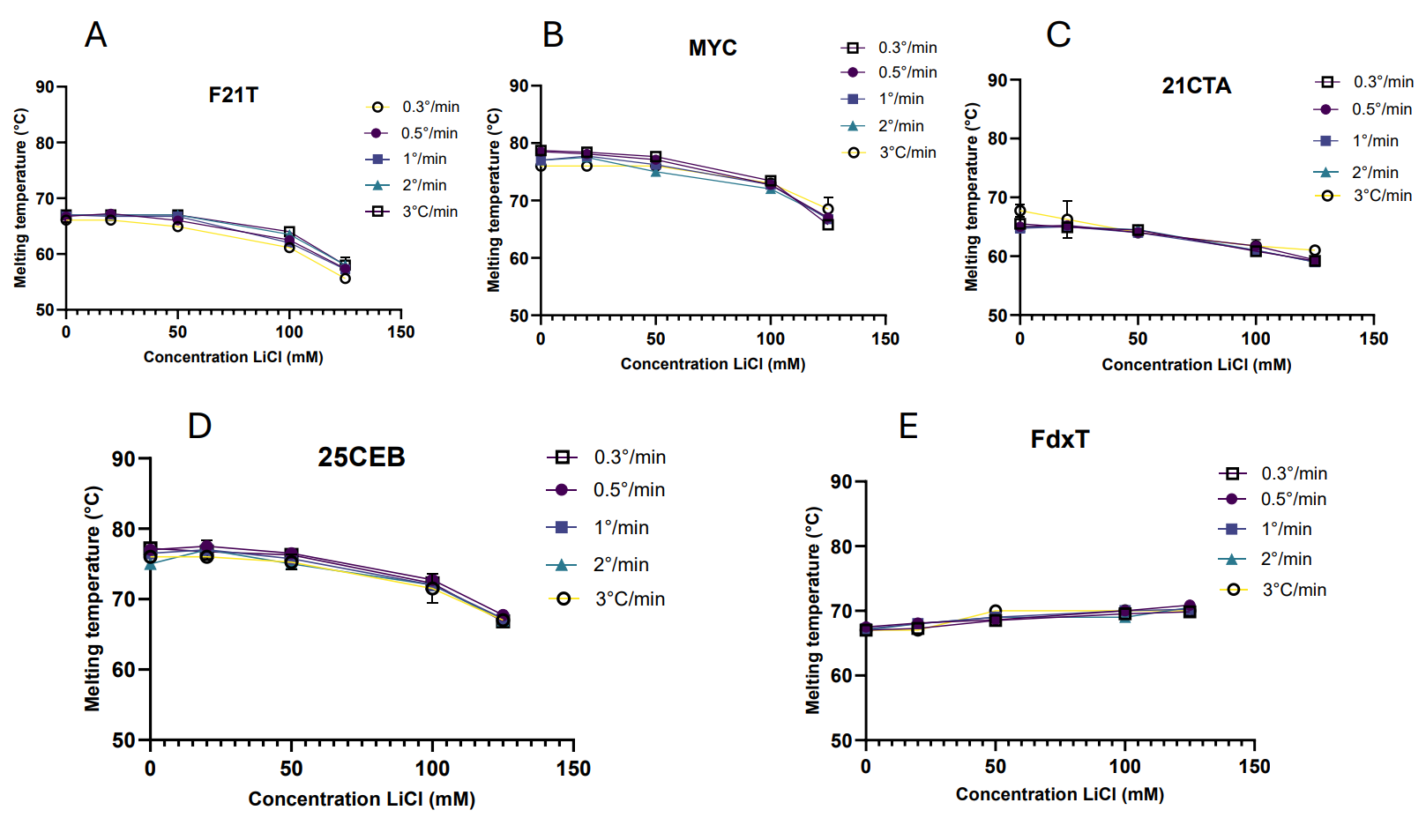
**

**
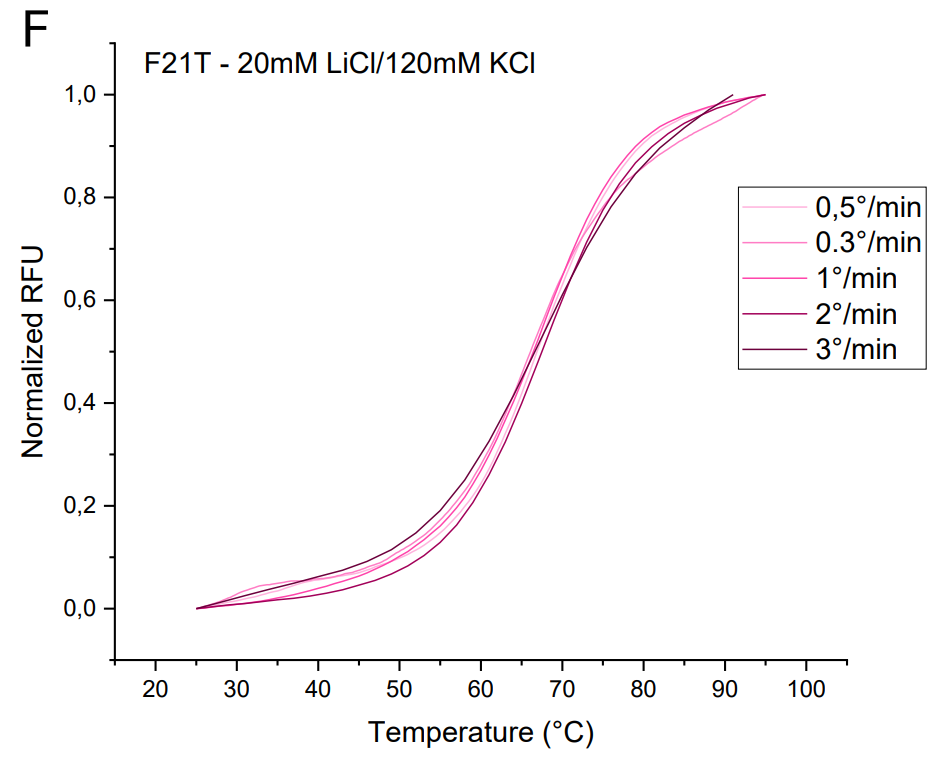
**

**Figure S3**. Melting temperature plotted for each condition **(A-E)** and for 4 G4s and one duplex, at different scanning rates (0.3 – 3 °C/min). Example of the curves obtained at the different scanning rates with F21T **(F)** and in 10 mM licaco, pH 7.2 supplemented with 20 mm LiCl and 120 mM KCl.

**
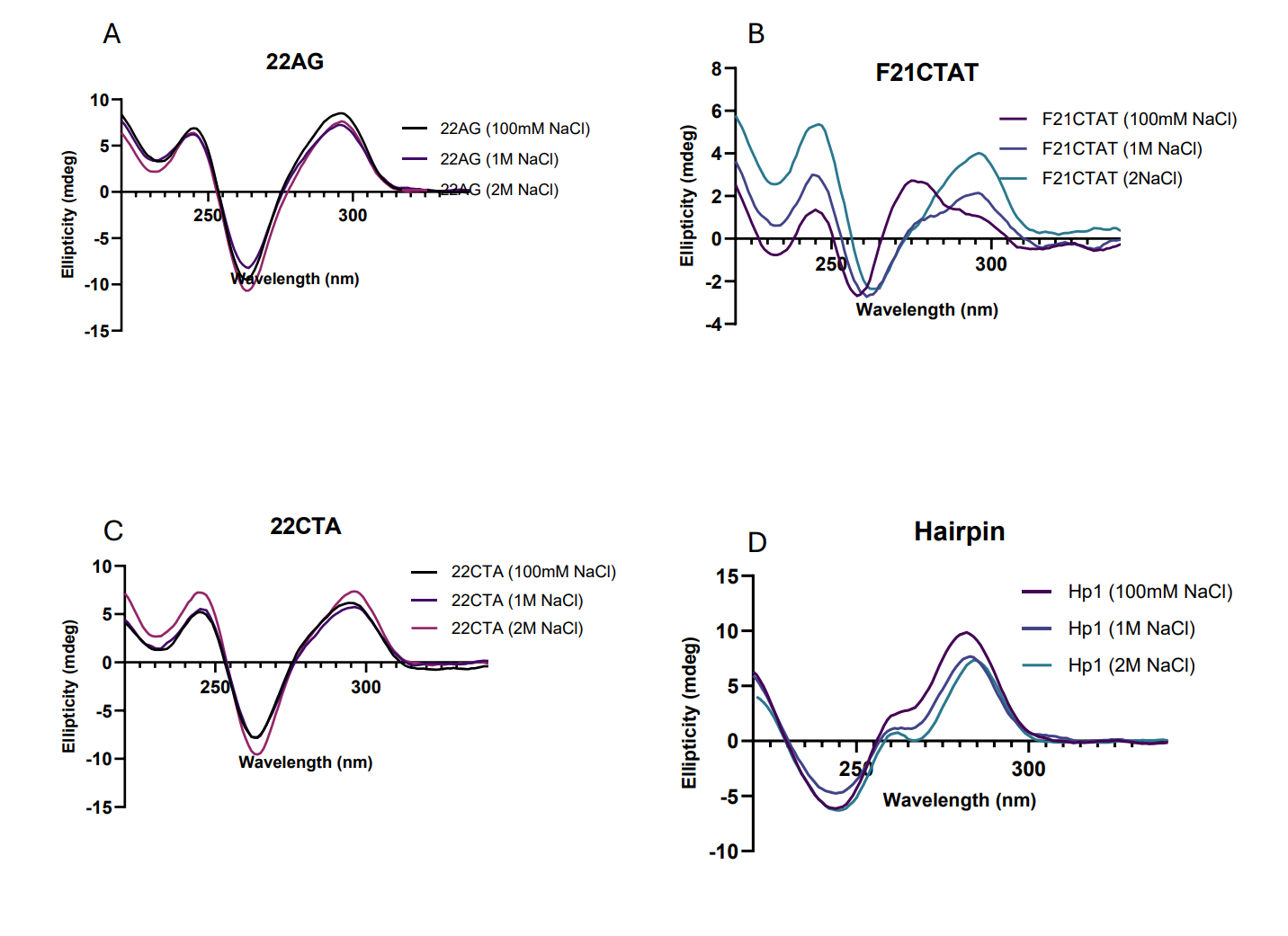
Figure S4**

**Figure S4**. Circular dichroism showing the effect of the increase in ionic strength, for non-modified DNA **(A – C – D)** and one labeled FAM-TAMRA G4 **(B)**, in sodium conditions.

**Figure S5**


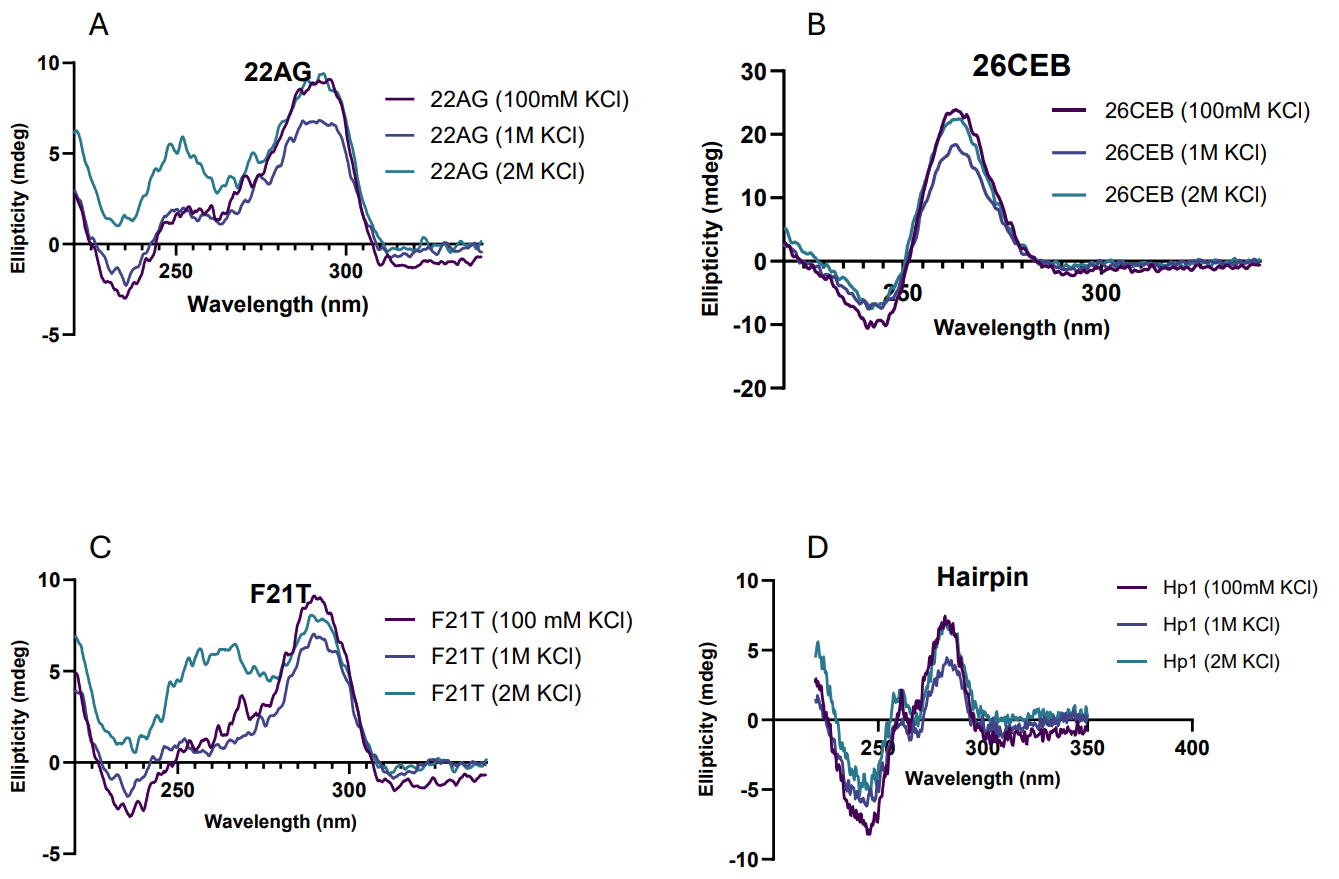


**Figure S5**. Circular dichroism showing the effect of the increase in ionic strength, for non-modified DNA **(A – B – D)** and one labeled FAM-TAMRA G4 **(C)**, in potassium conditions.
